# Validity of Hololens Augmented Reality Head Mounted Display for Measuring Gait Parameters in Healthy Adults and Children with Cerebral Palsy

**DOI:** 10.3390/s21082697

**Published:** 2021-04-11

**Authors:** Anne-Laure Guinet, Guillaume Bouyer, Samir Otmane, Eric Desailly

**Affiliations:** 1 Pôle Recherche & Innovation, Fondation Ellen Poidatz, 77310 Saint-Fargeau-Ponthierry, France; eric.desailly@fondationpoidatz.com; 2 IBISC Lab, University Paris-Saclay, University Evry, 91020 Evry, France; guillaume.bouyer@univ-evry.fr (G.B.); samir.otmane@univ-evry.fr (S.O.)

**Keywords:** spatiotemporal gait parameters, augmented reality, wearable device, cerebral palsy, concurrent validity

## Abstract

Serious games are a promising approach to improve gait rehabilitation for people with gait disorders. Combined with wearable augmented reality headset, serious games for gait rehabilitation in a clinical setting can be envisaged, allowing to evolve in a real environment and provide fun and feedback to enhance patient’s motivation. This requires a method to obtain accurate information on the spatiotemporal gait parameters of the playing patient. To this end, we propose a new algorithm called HoloStep that computes spatiotemporal gait parameters using only the head pose provided by an augmented reality headset (Hololens). It is based on the detection of peaks associated to initial contact event, and uses a combination of locking distance, locking time, peak amplitude detection with custom thresholds for children with CP. The performance of HoloStep was compared during a walking session at comfortable speed to Zeni’s reference algorithm, which is based on kinematics and a full 3D motion capture system. Our study included 62 children with cerebral palsy (CP), classified according to Gross Motor Function Classification System (GMFCS) between levels I and III, and 13 healthy participants (HP). Metrics such as sensitivity, specificity, accuracy and precision for step detection with HoloStep were above 96%. The Intra-Class Coefficient between steps length calculated with HoloStep and the reference was 0.92 (GMFCS I), 0.86 (GMFCS II/III) and 0.78 (HP). HoloStep demonstrated good performance when applied to a wide range of gait patterns, including children with CP using walking aids. Findings provide important insights for future gait intervention using augmented reality games for children with CP.

## 1. Introduction

Cerebral Palsy (CP) is the most common cause of childhood disability, affecting 17 million people worldwide [1,2]. CP describes a group of permanent disorders of the development of movement and posture, causing activity limitation, which are attributed to non-progressive disturbances that occurred in the developing fetal or infant brain [3]. Children with CP have different functional abilities, classified from Gross Motor Function Classification System (GMFCS) Level I (can walk and climb stairs without using hands for support) to Level V (impaired in all areas of motor function). In addition, gait in children with CP is characterized by a slower speed, a shorter-step length, a lower cadence, and more time spent in double support [4,5,6]. The natural history in children with CP is a gradual decline in ambulatory function as children grow and age, in particular during adolescence [7,8].

To reverse this trend, physiotherapists propose overground or treadmill-based gait training, with varying body weight support [9]. Functional gait training includes many different interventions and level of intensity to improve walking capacity. Grecco et al. highlighted the fact that rigorous gait training protocol, performed at the aerobic threshold with increasing intensity, was the key point to improve endurance, walking speed and daily functional performance [10]. But both for adults and children, with multiple repetition of the same task, the loss of motivation can decrease treatment adherence.

The application of technology in the field of rehabilitation is becoming increasingly popular. Within the range of choices available to therapists and patients, virtual/augmented reality (VR/AR) systems are feasible, effective and have positive effect on compliance with therapy and motivation of the patient [11,12]. These systems promote multi-sensory stimulation and user interaction, and can deliver feedback in real-time that could enhance motor learning and skill acquisition [13,14]. A meta-analysis by Chen et al. based on the ICF (International classification of functioning, disability and health [15]) concluded that VR/AR had a large effect size for the activity component (including ambulation function) for children with CP [16]. For example, after VR treadmill training, the distance travelled in the 2-Minutes Walk Test increased significantly, from 54.83 to 116.07 m in the VR group in children with CP [17].

Thanks to the miniaturization of the devices and the commercial development of affordable products, wearable technology-assisted devices appear to be an interesting tool for gait rehabilitation in overground condition (i.e., gait training without treadmill) [18,19,20,21]. Among existing tools, AR technology does not fully immerse the user in a simulated environment but superimpose virtual elements over the real-world. For example, Microsoft Hololens is an AR Head Mounted Display (HMD) which includes optical and inertial sensors for position and orientation tracking. It allows patients to walk with their walking aids and augments real environment with visual and auditory feedback. These features offer the possibility to develop a serious game based on motor learning theories to improve walking rehabilitation with gait pattern recognition.

Today, there is no AR system or serious game designed for overground gait training, including a rigorous protocol based on the patient’s walking performance and abilities. One of the essential components of such a system is the real-time tracking of the spatio-temporal gait parameters in people with gait disorders. Several methods exist that use full kinematic captures [22,23,24] or lighter inertial sensors [25,26,27,28,29,30]. Two recent works exploit an AR HMD but do not involve children with CP [31,32]. In this paper, we present a new algorithm, called HoloStep, to compute spatiotemporal gait parameters of children with CP using only the head pose recorded with a Hololens AR HMD. It is based on the detection of peaks associated to initial contact event, and uses a combination of locking distance, locking time, peak amplitude detection with custom thresholds for children with CP. We have evaluated its concurrent validity (i.e., between-systems agreement) in healthy adults and in children with CP using different walking aids (GMFCS I-II-III). Gait parameters (speed, step length, cadence, step detection) were compared to a reference algorithm from Zeni [22] which uses pelvic and feet kinematics data extracted from a motion capture system. The objective of this study was therefore to develop and validate an AR HMD based algorithm providing gait spatiotemporal parameters, in a real environment, both in healthy and children with gait disorders. We assume that HoloStep is accurate enough to be used in clinical practice to provide feedback on gait performance to the patient through the AR HMD.

## 2. Related Work

Multiple methods exist for calculating spatiotemporal gait parameters, whether based on force plates or reflective marker systems [22,23,24]. However, these techniques require expensive equipment and are only applicable in a gait laboratory.

Wearable sensors based on inertial measurement units (IMUs) or accelerometers have been validated in both normal and pathologic gait to detect gait events both in controlled laboratory conditions [25,26,27,28] and in real-life behaviour [29,30]. Using tri-axial accelerometer, Zijlstra et al. developed an algorithm predicting spatiotemporal gait parameters with trunk acceleration data for healthy participants, but step length and speed were underestimated both in overground and treadmill condition [25]. Trojaniello et al. have tested five methods for the estimation of gait events and temporal parameters from the acceleration signals of a single IMU. Data were acquired from healthy participants. Some methods estimate step time (i.e., the time between two consecutive initial contacts) and stride time (i.e., one complete gait cycle) to determine spatial parameters such as the step length. Some methods require the determination of both initial contacts (IC) and foot contacts (FC), and two methods associate acceleration signals and physical characteristics of gait to identify gait events [27]. They concluded that all methods are acceptable for clinical use (mean error in estimating stride time and step time are non-significant, maximal percentage of error is 2 to 4% for stride time and 2 to 8% for step time). McCamley at al. used vertical acceleration from IMU and means of continuous wavelet transform to detect foot contacts (initial and final). They reported an average error of 0.02 s and 0.03 s representing 2% and 3% of mean stride duration [28]. Storm et al. have compared two algorithms to determine temporal gait parameters based on two shank-worn IMUs or a single waist-worn IMU in free-living condition. IC and FC were detected inside predefined search windows. Then, the IC is identified as the instant of minimum angular velocity in the sagittal plane between the beginning of the IC search window and the instant of maximum anterior-posterior acceleration. The FC is identified as the instant of minimum anterior-posterior acceleration in the FC search window. For the second method using single waist-worn IMU, a first Gaussian continuous wavelet transformation is applied to the vertical acceleration signal, and the minima are identified as the IC timings. Results showed that the stride and step time absolute errors recorded using these methods were higher during outdoor free walking but generated only a small increase in percentage error (6 to 9 ms for stride time, and 9 to 14 ms for step time). Step length calculation was not assessed [29]. These previous studies concerned only healthy participants.

In a study including people with gait disorders, two magneto-inertial units including a tri-axial accelerometer, a tri-axial gyroscope and a tri-axial magnetometer (MIMUs) were fixed to the malleoli for the determination of both temporal and spatial parameters [26]. The gait events were detected using a specific period within which no gait events were expected and additional conditions also had to be satisfied. This complex algorithm was adapted to pathologic gait patterns to limit the risk of extra and missed gait events detection. Neither missed nor extra gait events were observed. Percentage of absolute error in estimating stride length was excellent both for hemiparetic people (3%), Parkinson’s disease people (2%) and choreic people (2%), both at comfortable speed and higher speed.

In children with CP-GMFCS I-II (i.e., less affected), some previous studies have demonstrated acceptable validity of accelerometry to detect mobility-related metrics, such as the total number of steps per day, walking distance [33,34,35], and cadence [36]. Sala et al. evaluated the accuracy of the wrist-based Fitbit Flex and the hip-based Fitbit One in quantitatively measuring the ambulation of children with CP, classified in GMFCS levels I to III, in a clinical setting. Participants were children with CP using different walking aids: any assistive device (n = 28), a posterior rollator (n = 7), one forearm crutch (n = 3), and two forearm crutches (n = 3). They demonstrated that wrist-based was not accurate for counting steps (range of errors between −484 to 35 steps). They reported better results for hip-based device (range of errors between −52 to 6). They concluded that for people having reduced mobility (walked slowly, took small steps, and used a rollator), the step counts for a hip-based and a wrist-based Fitbit must be considered with prudence [33]. These devices did not provide step length and gait speed. Other study assessed the accuracy in distance walked and step count of two commercial devices: the Minimod combining three accelerometers and the AMP (inertial sensors). Participants were diplegic CP and typically developing children. When the walked distance increased, both devices became less accurate and showed greater underestimation of actual distance walked and step count, steps differences were as high as 40 [34]. Both studies showed that commercial devices using a standard algorithm for detecting temporal gait parameters were not suitable for patients with gait disorders. Recently, Paraschiv-Ionescu et al. have developed a custom-made algorithm based on detection of peaks associated to heel-strike events, and included several processing stages such as peak enhancement and selection of the steps-related peaks using heuristic decision rules. They used the norm of trunk acceleration signals from Physilog4^®^ (device including 3D accelerometer, 3D gyroscope, 3D magnetometer and barometer) worn on the trunk. They showed a very good sensitivity, specificity and precision for detection of locomotion period: between 86% to 97%. But, they highlighted that for short period of locomotion and/or if the gait pattern is unsteady with high variability, the error can be important [36]. Moreover, IMU has been successfully used as a tool for diagnosing pathological gait providing estimation about joint kinematics parameters. Glowinski et al. developed a new algorithm combining discrete Fourier transform (DFT) and continuous wavelet transform (CWT). Based on IMU, they identified significant differences in knee flexion during gait in patient with lumbar discopathy [37].

Globally, results indicate that adaptive and custom algorithm is suitable for calculating spatio-temporal gait parameters in people with gait disorders. But these techniques, despite their great robustness, have potential drawbacks for coupling them with AR/VR systems in clinical context. The need for multiple devices to maximize accuracy, the difficulty in synchronizing with AR/VR systems and the complexity of the user’s equipment mean that the system is not “plug and play”. A simpler configuration, with a single device, could allow for wider clinical application.

To our knowledge, there are very few studies using an AR HMD (Hololens) for calculating spatio-temporal gait parameters. As a preliminary study, Guinet et al. showed that the accuracy of the Hololens was sufficiently high to evaluate the position of the user’s head, without spatial drift, in comparison to MOCAP system. They found an absolute errors between 55 to 250 mm in all 3 planes [38]. Using the same device, Geerse et al. tested a method using head vertical maximal position to estimate foot step location [32]. This algorithm has shown a good test-retest reliability and a good concurrent validity at different walking speeds for healthy participants and for people with Parkinson’s disease (PD). Still, they observed significant differences between their method and the reference for walking speed, step length and cadence. They also had measurement biases increasing with faster instructed walking speeds. Finally, Ju et al. have proposed a machine learning approach for detecting whether a healthy subject was touching the ground with the left or right foot while walking [31]. This method required the sound recording of footsteps in real-time, that is impossible in clinical use because of the ambient noise. Moreover, this algorithm was not validated for people with gait disorders.

## 3. Materials and Methods

### 3.1. Participants

Thirteen healthy adults and sixty-two children with CP were included. Healthy adults had to be 18 years or older and normal or corrected vision. Subjects were recruited at the Poidatz Rehabilitation Center adjacent to the gait lab. The inclusion criteria for people with CP were an age between 10–18 years, a Gross Motor Function Classification System (GMFCS) [39] I to III. Children for whom a gait analysis test was planned were invited to participate in this study when they met the inclusion criteria. Written consent was previously obtained from each child’s parent or guardian and assent from each child to collect and use their clinical data. Children were divided in two groups: Group 1: Children with CP walking without walking aids (N = 32) (i.e., GMFCS I), Group 2: Children with CP walking with crutches or posterior walker (N = 30) (i.e., GMFCS II/III). Characteristics of children included were summarized in Table 1. The study was conducted according to the guidelines of the Declaration of Helsinki, and approved by the National Ethics Committee (see Institutional Review Board and Informed consent statement below). Test session was held in July 2020.

### 3.2. Gait Analysis Systems

#### 3.2.1. MOCAP System

The gait laboratory used a fifteen-camera VICON system (8 MX 20, 5 T 40, 2 T 160) (PluginGait marker set, VICON, Oxford Metrics, UK). Data were collected from markers placed on the headband (4), pelvic (4) and feet (6). Data were recorded at 100 Hz and filtered using a real-time 2nd order low-pass Butterworth filter (with a cutoff frequency of 6). VICON system was considered as a reference for gait analysis and spatiotemporal gait parameters calculation. Hereafter in this article, VICON system has been called MOCAP.

#### 3.2.2. Hololens AR HMD

The HoloStep algorithm has been written in C# language. It was a part of the AR application developed with Unity 2019.2.8f1 (64-bit) using Mixed Reality Toolkit version 2 for Microsoft Hololens HMD. This version of AR application did not contain any hologram in order to be comparable to MOCAP. Data were recorded at 100 Hz and filtered using a real-time 2nd order low-pass Butterworth filter (with a cutoff frequency of 6). This application has been deployed in Microsoft Hololens version 1 [40]. In the following, Microsoft Hololens is called AR HMD.

### 3.3. The HoloStep Computational Method

#### 3.3.1. Step Detection

**Figure 1:**
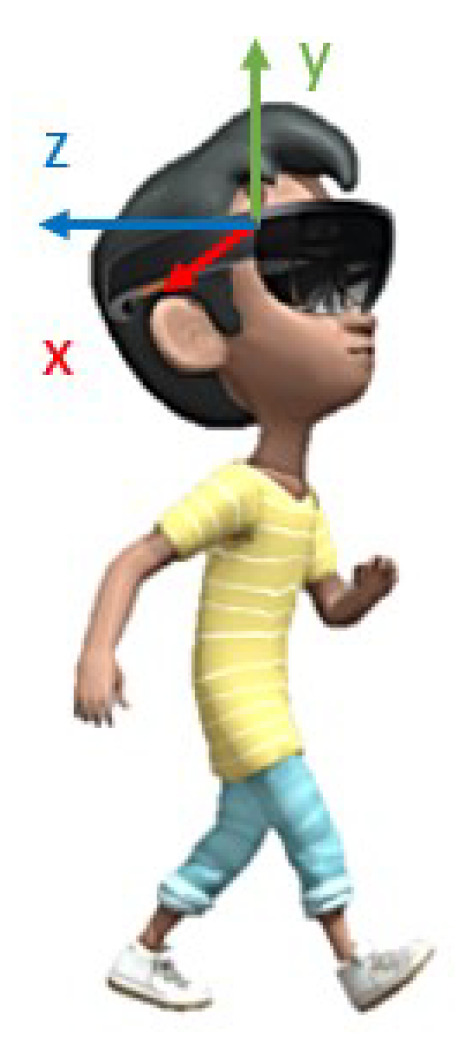
Spatial coordinate system of the AR HMD is (0; $\vec{x}$; $\vec{y}$; $\vec{z}$).

When the application starts, the spatial coordinate systems of the HMD were right-handed, which means that the positive *X*-axis points right, the positive *Y*-axis points up (aligned to gravity) and the positive *Z*-axis points towards you (Figure 1) [40].

The data acquisition rate was 100 Hz. At each frame, HoloStep calculates: Time (s), Position ($x_{H}$, $y_{H}$, $z_{H}$) (m), Filtered position ($x_{F}$, $y_{F}$, $z_{F}$) (m), Step detected (*Boolean*), Step length (m) and Walking distance from the beginning of the trial (m). These data are stored in memory for direct use in the AR application, and logged in a .csv file for later analysis.

The initial position $P_{t_{0}}$ of the AR HMD was:

$$P_{t_{0}}={\left(0,AR\;headset\;y\;level,0\right)}_{t_{0}}$$

Then at any time *t* the AR HMD position $P_{t}$ was given in this reference frame:

$$P_{t}={\left(x_{H},y_{H},z_{H}\right)}_{t}$$

The position signal *P* was then filtered using a second-order zero-lag Butterworth low pass filter with a 6 Hz cut-off frequency to get $P_{F}$. Minimum peaks were detected using $y_{F}$ the filtered AR HMD vertical position signal.

Each gait cycle was divided into two phases: stance and swing. Stance consisted of the entire time that a foot was on the ground, starting with an initial contact (IC) when the foot touched the ground, and ending with a toe off (TO) when the foot left the ground. Swing corresponded of the entire time that a foot was in the air, starting with TO and ending at the next IC. The principle of detection used by HoloStep was based on the fact that during walking, the body displacement is pseudo-periodic. The body slightly leaned to the left/right side. It created a characteristic variation on *x*-axis. Moreover, at each IC (i.e., when the foot was touching the ground), the body was at a lower position on *y*-axis. Therefore, when the user was leaning to the left (*x* min) and the body was at a lower position (*y* min), the left foot of the user started to touch the ground. The similar situation happened on the other side (*x* max and *y* min).

**Figure 2:**
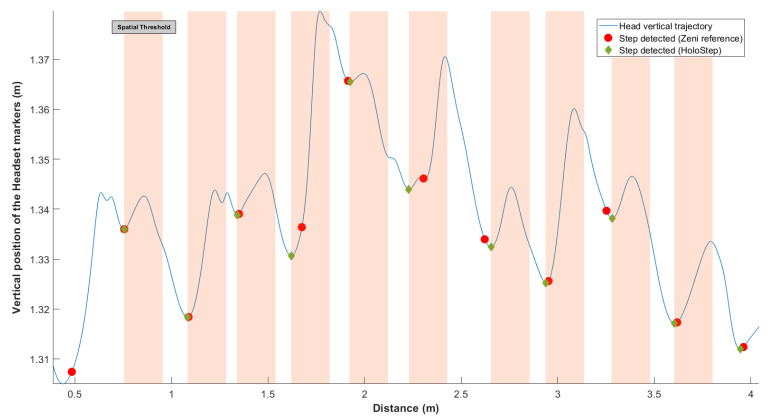
Representation of the locking distance threshold. In blue, vertical head position $y_{F}$ of the user wearing the AR HMD over the walkway. The green diamonds were IC detected with HoloStep. The red circle were IC detected with Zeni algorithm reported to $y_{F}$. The orange bands were the locking distance threshold (minimum peaks ignored by HoloStep).

**Figure 3:**
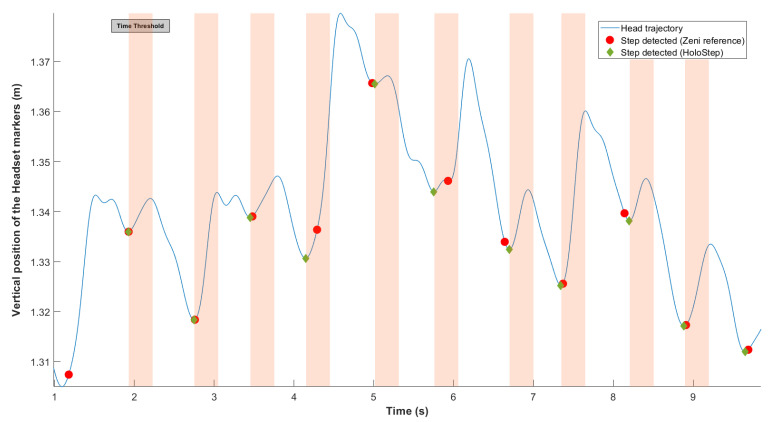
Representation of the locking time threshold. In blue, vertical head position $y_{F}$ of the user wearing the AR HMD over the walkway. The green diamonds were the IC detected with HoloStep. The red circle were IC detected with Zeni algorithm reported to $y_{F}$. The orange bands were the locking time threshold (minimum peaks ignored by HoloStep).

HoloStep was developed using a combination of locking distance [41], locking time [42,43] and peak amplitude detection with custom thresholds for children with CP. In each window, the minimum peak position on $y_{F}$ signals was used to detect initial contact IC. In order to define if the distance between two peaks should be considered as a real step, three conditions were checked:

- First, the *locking distance* was defined: new IC was considered only if the distance between two IC was greater than this threshold (Figure 2). In order to make Holostep the most suitable for children with CP, we reviewed a separate data set from the gait analysis of 188 children with CP in a specialised laboratory. The mean distance between two IC was 44.31 cm, with SD = 11.87. We have set the locking distance at 20 cm (rounded down of mean−2×SD).

- Second, the *locking time* was defined: new IC was considered only if the time between two IC detected was greater than this threshold (Figure 3). As before, after analysis of the specific data set for children with CP, mean time between two IC was 56.01 ms, with SD = 12.54. The locking time was set to 30 ms (rounded down of mean−2×SD).

- Third, the *peak amplitude* threshold was defined: the minimum difference required between previous maximal ${\left(P_{y}\right)}_{f}$ and current minimal ${\left(P_{y}\right)}_{f}$. As before, after analysis of the specific data set for children with CP, mean peak amplitude detection was small at 0.3 cm. This is the value we have retained for the peak amplitude threshold.

#### 3.3.2. Step Length and Walking Distance

**Figure 4:**
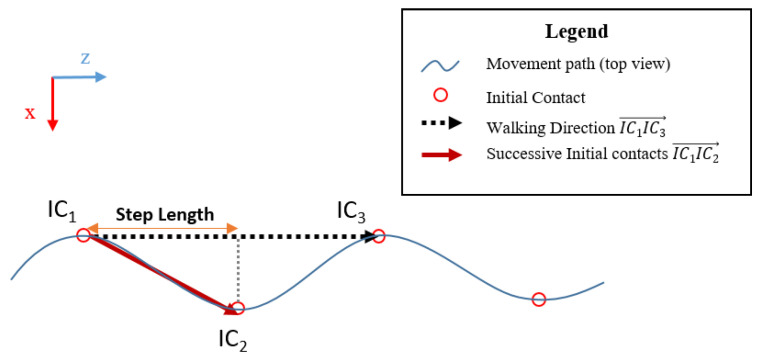
Representation of Step Length in the coordinate system of the AR headset (0; $\vec{x}$; $\vec{y}$; $\vec{z}$).

After detecting 2 consecutive steps, step length (SL) was the dot product between walking direction $\vec{IC_{1}IC_{3}}$ and two successive initial contacts $\vec{IC_{1}IC_{2}}$ (Figure 4):

$$\vec{IC_{1}IC_{2}}\cdot \vec{IC_{1}IC_{3}}=IC_{1}IC_{2}\times IC_{1}IC_{3}\times \cos\left(\hat{IC_{2}IC_{1}IC_{3}}\right)$$

Finally, the total walking distance was the sum of the successive step lengths.

**Figure 5:**
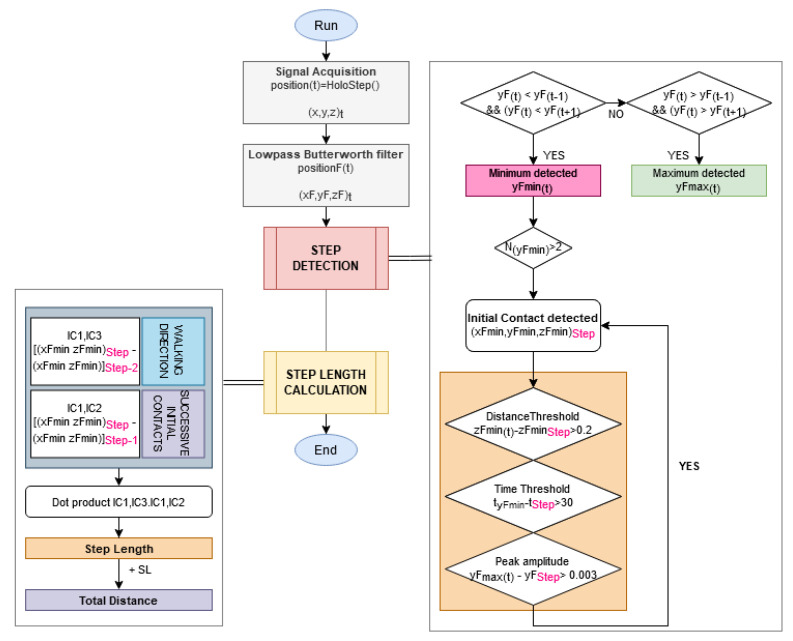
Flow chart of the proposed method HoloStep for step detection and step length calculation of children with CP.

Figure 5 summarises the flow chart of the HoloStep algorithm.

### 3.4. Experimental Procedure

Participants were instructed to walk at a comfortable speed in a straight line along an 8-m path in the gait lab. Data were collected for three successful trials.

### 3.5. Data Processing

The user’s head position was measured with the AR HMD and with MOCAP using 4 reflective markers placed on the AR HMD. Pelvic and feet position were measured with MOCAP using reflective markers. Dataset was processed using two different algorithms:

- Reference: Zeni algorithm using a set of pelvic and feet markers calculating spatiotemporal gait parameters with high accuracy [22];
- Challenger: HoloStep algorithm using head pose.

From HoloStep algorithm (C#) deployed in the AR HMD, user’s position ($x_{H}$, $y_{H}$, $z_{H}$), user’s position filtered ($x_{F}$, $y_{F}$, $z_{F}$), walking speed, step length, number and timing of step detected were extracted in .csv format. From MOCAP, user’s position filtered was extracted from the reflective markers placed on the AR HMD ($x_{G}$, $y_{G}$, $z_{G}$), walking speed, step length and number of step were calculated using Zeni algorithm. The AR HMD signals were synchronized to the MOCAP signals by an automatic time shifting procedure using a local minimum detection. An Iterative Closest Point (ICP) algorithm was used to align the 3D positions given by the two systems minimizing the distance between them, using geometric transformations (rotations *R* and translations *T*) [44,45]. The ICP algorithm had two steps: The first step consisted of determining the correspondence pairs $\left(\vec{p},\vec{m}\right)$ from two data sets *H* and *G*. The aim was to find for each point *p* in *H* its closest point in *G*. The second step was to apply a transformation (*R* and *T*) in order to minimize the distance between the correspondence pairs:

$$E\left(R,T\right)=\frac{1}{N_{H}}\sum_{i=1}^{N_{H}}{∥\begin{array}{c} G_{i}-RH_{i}-T \end{array}∥}^{2}$$

with $H=(x_{H},y_{H},z_{H})$ and $G=(x_{G},y_{G},z_{G})$ the corresponding points, from AR HMD and from MOCAP respectively. These two steps were repeated until the error was below a given threshold or until the maximum number of iterations was reached. Data processing was performed with MATLAB 2019a.

### 3.6. Statistical Analysis

Bland and Altman analysis was performed to compare walking speed, step length and cadence between the two methods given the bias $\bar{d}$ (the mean of the differences between the two methods) and the limits of agreement (LoA). The Intraclass Correlation Coefficient for absolute agreement among measurements, also known as criterion-referenced reliability, was calculated (ICC(A,1)) [46]. The Pearson correlation coefficient *r* and determination coefficient $r^{2}$ were calculated to compare the two methods.

The two-sample t-test was done to compare means of the two methods for walking speed, step length and cadence. The 5% significance level was used to reject the null hypothesis. The confusion matrix allowed visualization of the performance of HoloStep algorithm: sensitivity (true positive rate), specificity (true negative rate), accuracy (true negative and positive rate) and precision (positive predictive value) of step detection for HoloStep algorithm, for each group, were calculated. Each row of the matrix represented the instances in the challenger class (HoloStep), while each column represented the instances in the reference class (Zeni).

## 4. Results

### 4.1. Healthy Participants

For the 13 healthy participants, the total number of IC detected was 65 with Zeni and 63 with HoloStep. The HoloStep algorithm ignored 2 IC (false negative) for 2 participants. Sensitivity, specificity, accuracy and precision of HoloStep algorithm were excellent (Table 2). The Bland and Altman analysis shown a bias $\bar{d}$ = 0.054 m for step length and 0.035 m/s for walking speed. The mean difference between the two algorithms for all variable were not significant. The ICC coefficients were excellent for walking speed (ICC = 0.973) and good for step length and cadence (ICC = 0.778 and 0.534, respectively). The mean difference for step length between Zeni and HoloStep was 5.6 cm (Table 3).

### 4.2. Children with CP

**Figure 6:**
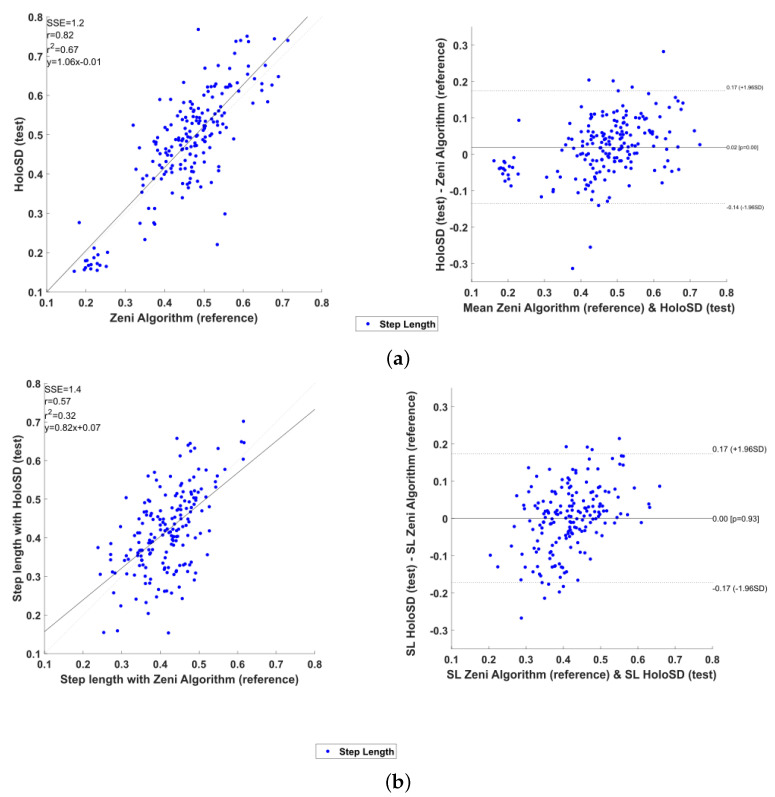
Statistical analysis graphics for children with CP. Left: Linear Distribution of step length between the two algorithms. Right: Bland and Altman plot representing step length. A bolt horizontal line representing the bias. Additional dotted horizontal lines, limits of agreement, are added to the plot at $\bar{d}\pm 1.96$ SD. (**a**) Children with CP—Group 1 (**b**) Children with CP—Group 2.

**Figure 7:**
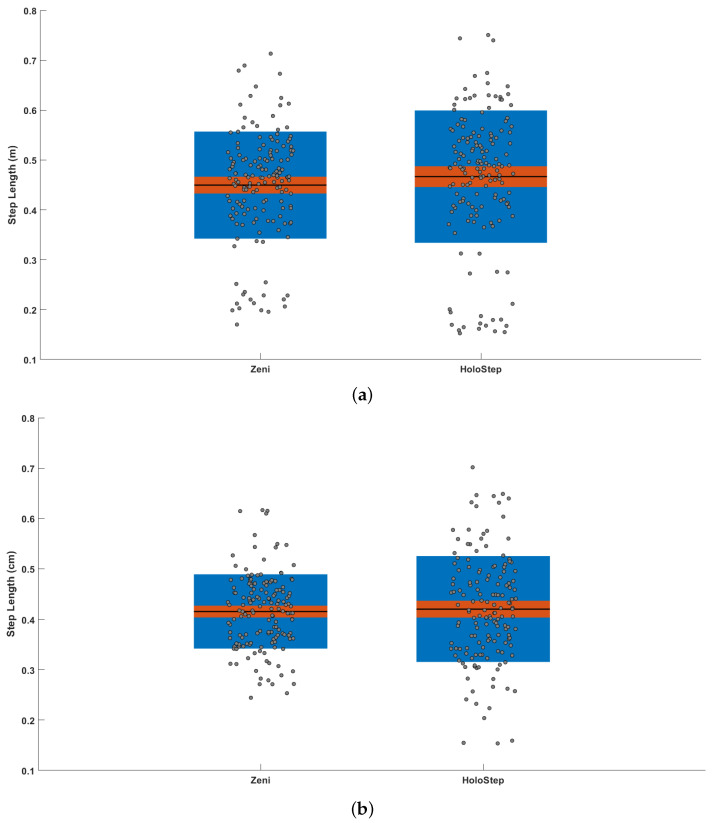
Difference between HoloStep and Zeni algorithm. Box plot visualizing raw data along the mean, 95% confidence interval, and 1 SD. (**a**) Children with CP—Group 1 (**b**) Children with CP—Group 2.

For the 32 participants from group 1 (children with CP walking without aids/GMFCSI), the total number of IC detected was 194 with Zeni and 195 with HoloStep. The HoloStep algorithm ignored 6 IC (false negative) and added 7 false IC (false positive) for 7 different patients. Sensitivity, specificity, accuracy and precision of HoloStep algorithm were excellent (Table 2). The Bland and Altman analysis shown a bias $\bar{d}$ = 0.017 m for step length and 0.018 m/s for walking speed (Figure 6a). The mean difference between the two algorithms for all variable were not significant (Figure 7a). The ICC coefficients were excellent for step length and walking speed (ICC = 0.922 and 0.996 respectively) and good for cadence (ICC = 0.642). The mean difference for step length between Zeni and HoloStep was 2.6 cm (Table 3).

For the 30 participants from group 2 (children with CP walking with aids/GMFCSII-III), the total number of IC detected was 184 with Zeni and 185 with HoloStep. The HoloStep algorithm ignored 2 IC (false negative) and added 3 false IC (false positive) for 3 different patients. Sensitivity, specificity, accuracy and precision of HoloStep algorithm were excellent (Table 2). The Bland and Altman analysis shown a bias $\bar{d}$ = 0.005 m for step length and 0.018 m/s for walking speed (Figure 6b). The mean difference between the two algorithms for all variable were not significant (Figure 7b). The ICC coefficients were excellent for step length and walking speed (ICC = 0.863 and 0.990 respectively) and good for cadence (ICC = 0.625). The mean difference for step length between Zeni and HoloStep was 4 cm (Table 3).

## 5. Discussion

This study is the first to describe and assess the validity of a custom algorithm deployed in the Hololens AR HMD to calculate spatiotemporal gait parameters both in healthy participants and children with CP. The results of our study suggest that HoloStep algorithm using Hololens AR HMD calculate spatiotemporal gait parameters with sufficient accuracy even in people with gait disorders using walking aids.

### 5.1. Comparison with Other Methods

HoloStep performance for speed calculation was comparable to those obtained with an IMU. For ex., Zijlstra et al. obtained a mean difference between predicted and real speeds below 0.05 m/s [25]. HoloStep had a mean difference from 0.018 m/s to 0.035 m/s between groups. For foot contacts detection, the sensitivity across various methods using IMU varied between 81% and 100% [27], whereas HoloStep was between 97% to 99%. For step length, the mean absolute error in estimating stride length for adult with gait disorders varied from 1.8 cm for hemiparetic to 2.6 cm for choreic people. HoloStep error varied from 2.6 cm to 4 cm for children with CP. This gap could be explained by the use of a posterior rollator which led to the detection of false steps. HoloStep using AR HMD obtained better results than other commercial devices like the wrist-based FitBit Flex and the hip-based FitBit One in quantitatively measuring the ambulation of children with CP [33]. In this study, mean absolute error in number of steps detected for children varied from 6 to 17 depending on the used walking aids. For HoloStep, those parameters varied from 2 to 7. Paraschiv-Ionescu et al. have developed a robust algorithm that used data from IMU device worn on lower back (L5 vertebrae) to calculate gait parameters. Performance metrics (sensitivity, specificity and precision) were excellent, from 0.90 to 0.98 for children with CP. But, this algorithm could not be used in the AR HMD because it used the norm of trunk acceleration signals.

Recently, Geerse et al. proposed a method to calculate spatiotemporal gait parameters using a Hololens AR HMD and compared this method to the reference Interactive Walkway System (IWS). They found that ICC were excellent for between-systems agreement for walking speed, step length and cadence for healthy adults and people with Parkinson’s Disease (PD) (ICC > 0.92). But limits of agreement obtained with Bland-Altman analysis were quite narrow. Still, walking speed and step length were underestimated with biases increasing with faster walking speeds (min bias of 0.6 for people with PD to 3.1 for healthy people walking faster) [32]. They also found statistical difference between step length measured with Hololens and IWS (*p* < 0.05). These between-systems biases were justified by the authors because of the drift in tracking and deviations in the map (caused by the Hololens AR HMD spatial mapping component). Using data available in Supplementary Materials, we have calculated step length with HoloStep. Using population-best matched thresholds, we have found no statistical difference between step length calculated with HoloSD and IWS, and HoloStep seemed to have better results on the biases (min bias of 0.07 for people with PD to 1.9 for healthy walking faster). These results suggest that the use of custom thresholds enhances the calculation of spatiotemporal gait parameters, but a stronger method to compare these two algorithms is necessary to conclude.

Sun et al. have developed two AR-based automated functional mobility test using Hololens AR HMD: Sit To Stand (STS) and Time Up and Go (TUG). In comparison with reference inertial sensor (Opal, APDM), vertical kinematic data (displacement, velocity and acceleration) shown a bias less than 0.02 s for STS and 0.13 s for TUG, with range of error within ±0.8 s. Correlation coefficient for kinematic measurement agreement between Hololens and reference sensors were from 0.74 to 0.99 [47]. We obtained similar results with HoloStep for kinematic measurement.

### 5.2. Gait Detection for Children with CP

Children with CP present alteration of dynamic stability during gait because of deficits in balance and postural control. Hsue et al. demonstrated that children with CP showed significantly larger vertical and medio-lateral displacements of the Center of Mass than TD group. But, the trajectories have the same shape (sinusoidal pattern) both for TD group than children with CP. In vertical directions, the Center of Mass reached a maximum peak at mid-stance, and a minimum at the end of terminal stance (when the two feet are in contact with the ground). It was interesting to observe that the minimum peaks were shifted for children with CP (4% of gait cycle after) [48]. When children have an asymmetrical gait (this is often the case for children using crutches), this pattern was not that regular: amplitude of the first peak (more affected side) is higher than the second [48]. Moreover, children with CP walking with crutches presented some small and inconsistent peaks on vertical axis. This variation of walking pattern conducted to a lot of false IC detection by existing algorithms. HoloStep used custom thresholds, based on the analysis of CP gait pattern, in order to minimize these bias. Eyes, head and chest orientation are strongly correlated when people walk with a fixed gaze direction [49]. The head and trunk trajectories are also linked during locomotion (signals have the same sinusoidal shape) [50]. These considerations encouraged to develop wearable technology fixed to the participant’s head [51] which is the case of a HMD.

### 5.3. HoloStep Limitations

HoloStep is based on the kinematics of the head, which requires special attention as it has 6 degrees of freedom and can have variable patterns. In sagittal plane, the variability of head displacement for children with CP is high during gait [52]. But, at initial contact, Heyrman et al. reported that ICC within and between session were above 0.7 (comparable to the thorax kinematics) [52]. In our study, children were asked to look straight ahead during walking tests to minimize head movement, which could be considered as non spontaneous gait. However, this constraint is relevant to the future use of the Hololens AR HMD, which will display holograms in front of the subject, encouraging them to keep their gaze on the horizon. Each child produced distinctive walking pattern, peak amplitudes could be different (regular, asymmetric, short, round...). Thus, a method based on individual signals could be better than predefined thresholds [41]. The IC detection could be more specific to the individual’s gait pattern. In the same way, the locking period threshold could be customized for one gait pattern. The use of machine learning method could contribute to improve step detection, and by extension step length [31]. Another limitation is that HoloStep has been tested only with the Hololens AR HM version 1. Using another device would probably require some adjustments to the thresholds used. Furthermore, it can be assumed that future versions of the various manufacturers’ HMD will further improve the robustness and accuracy of head pose capture.

## 6. Conclusions and Future Work

We have developed and evaluated a new algorithm called HoloStep to calculate spatiotemporal gait parameters using only the head pose provided by an augmented reality headset (Hololens). It is based on the detection of peaks associated to initial contact event, and used a combination of locking distance, locking time, peak amplitude detection with custom thresholds for children with CP. An experimental comparison with a reference algorithm based on full motion kinematics shown that HoloStep accurately detected foot contact, and calculated step length, total distance walked and gait speed both for children with CP and healthy participants.

Once the gait parameters have been obtained, another step before designing relevant rehabilitation exercises is to investigate feedback on gait performance in AR. We therefore started to model and evaluate different visual feedback on speed, and their effects on patients’ walking performance. We have also started the development of a serious game using a process framework that involves a multidisciplinary team and is inspired by existing methodologies [53,54]. This serious game includes sprint training sessions and adaptive feedback. We will start a larger clinical study to assess acceptance, usability [55] and therapeutic effects on children with CP.

Beyond this specific application, we believe that the HoloStep algorithm can be implemented in other serious games aimed at a wider range of different patients for motor rehabilitation purposes.

## Figures and Tables

**Table 1:** Characteristics of population included.

Characteristics	Healthy Participant (n = 13)	Children Group 1 (n = 32)	Children Group 2 (n = 30)
Age (mean)	35.9	12.6	12.3
Sex (F/M)	5/8	16/16	13/17
GMFCS	NA	I	II–III
Walking aids	No	No	Crutches (18)
			Posterior walker (12)

**Table 2:** Sensitivity, specificity, accuracy and precision of step detection with HoloStep algorithm for healthy participants and children with CP.

	Sensitivity	Specificity	Accuracy	Precision
Healthy participant (n = 13)	0.969	1.000	0.999	1.000
Children Group 1 (n = 32)	0.969	0.999	0.999	0.964
Children Group 2 (n = 30)	0.989	1.000	1.000	0.984
All CP children (n = 62)	0.979	1.000	0.999	0.974

**Table 3:** Concurrent validity for spatiotemporal gait parameters in healthy participants (HP) and in children with cerebral palsy (Group 1 and Group 2).

		MOCAP Zeni	Hololens HMD HoloStep				
		Mean +/− SD	Mean +/− SD	Bias (95% LoA)	t-statistics	ICC(A,1)	r corr
Walking Speed (m/s)	CP-Group 1	1.044 +/− 0.254	1.026 +/− 0.258	0.018 (−0.012 0.048)	t(30) = −0.28, *p* = 0.78	0.996	0.998
	CP-Group 2	0.667 +/− 0.180	0.648 +/− 0.174	0.018 (−0.017 0.053)	t(28) = −0.40, *p* = 0.69	0.990	0.995
	HP	1.277 +/− 0.199	1.242 +/− 0.197	0.035 (−0.026 0.096)	t(11) = −0.45, *p* = 0.66	0.973	0.988
Step Length (m)	CP-Group 1	0.488 +/− 0.090	0.514 +/− 0.105	0.017 (−0.106 0.140)	t(30) = 1.07, *p* = 0.29	0.922	0.885
	CP-Group 2	0.430 +/− 0.064	0.434 +/− 0.079	0.005 (−0.152 0.162)	t(28) = 0.18, *p* = 0.86	0.863	0.649
	HP	0.623 +/− 0.079	0.679 +/− 0.087	0.054 (−0.048 0.156)	t(11) = 1.74, *p* = 0.095	0.778	0.802
Cadence (steps/s)	CP-Group 1	1.980 +/− 0.272	1.838 +/− 0.267	0.142 (−0.249 0.534)	t(30) = −2.11, *p* = 0.29	0.642	0.726
	CP-Group 2	1.178 +/− 0.308	1.425 +/− 0.294	−0.247 (−0.589 0.095)	t(28) = 3.18, *p* = 0.86	0.625	0.833
	HP	1.908 +/− 0.196	1.808 +/− 0.231	0.0099 (−0.288 0.486)	t(11) = −1.18, *p* = 0.095	0.534	0.582

## Supplementary Materials

Supplementary materials are available online at https://www.mdpi.com/article/10.3390/s21082697/s1.

## Abbreviations

The following abbreviations are used in this manuscript:

AR	Augmented Reality
CP	Cerebral Palsy
FC	Foot Contact
GMFCS	Gross Motor Function Classification System
HMD	Head Mounted Display
HP	Healthy Participants
IC	Initial Contact
ICC	Intra-Class Coefficient
SD	Standard Deviation
SL	Step Length
TD	Typically Developing
TO	Toe Off
VR	Virtual Reality
